# Impact of epicardial adipose tissue volume on hemodynamically significant coronary artery disease in Chinese patients with known or suspected coronary artery disease

**DOI:** 10.3389/fcvm.2023.1088961

**Published:** 2023-03-21

**Authors:** Xiangbo Jin, Beibei Gao, Jiamin Zheng, Xueer Wu, Ning Zhang, Lijun Zhu, Xinyu Zhu, Jianchang Xie, Zhen Wang, Guoxin Tong, Jinyu Huang

**Affiliations:** ^1^ Department of Cardiology, Affiliated Hangzhou First People’s Hospital, Zhejiang University School of Medicine, Hangzhou, China; ^2^ Graduate School, Zhejiang Chinese Medical University, Hangzhou, China; ^3^ Graduate School, Wenzhou Medical University, Wenzhou, China; ^4^ Department of Cardiology, Ningbo Municipal Medical Center LiHuili Hospital, Ningbo, China; ^5^ Department of Radiology, Affiliated Hangzhou First People’s Hospital, Zhejiang University School of Medicine, Hangzhou, China

**Keywords:** coronary artery calcium, epicardial adipose tissue, fractional flow reserve, hemodynamically significant coronary artery disease, obstructive coronary artery disease, quantitative flow ratio

## Abstract

**Background:**

Epicardial adipose tissue (EAT) is directly related to coronary artery disease (CAD), but little is known about its role in hemodynamically significant CAD. Therefore, our goal is to explore the impact of EAT volume on hemodynamically significant CAD.

**Methods:**

Patients who underwent coronary computed tomography angiography (CCTA) and received coronary angiography within 30 days were retrospectively included. Measurements of EAT volume and coronary artery calcium score (CACs) were performed on a semi-automatic software based on CCTA images, while quantitative flow ratio (QFR) was automatically calculated by the AngioPlus system according to coronary angiographic images.

**Results:**

This study included 277 patients, 112 of whom had hemodynamically significant CAD and showed higher EAT volume. In multivariate analysis, EAT volume was independently and positively correlated with hemodynamically significant CAD [per standard deviation (SD) cm^3^; odds ratio (OR), 2.78; 95% confidence interval (CI), 1.86–4.15; *P *< 0.001], but negatively associated with QFR_min_ (per SD cm^3^; *β* coefficient, −0.068; 95% CI, −0.109 to −0.027; *P* = 0.001) after adjustment for traditional risk factors and CACs. Receiver operating characteristics curve analysis demonstrated a significant improvement in predictive value for hemodynamically significant CAD with the addition of EAT volume to obstructive CAD alone (area under the curve, 0.950 vs. 0.891; *P *< 0.001).

**Conclusion:**

In this study, we found that EAT volume correlated substantially and positively with the existence and severity of hemodynamically significant CAD in Chinese patients with known or suspected CAD, which was independent of traditional risk factors and CACs. In combination with obstructive CAD, EAT volume significantly improved diagnostic performance for hemodynamically significant CAD, suggesting that EAT could be a reliable noninvasive indicator of hemodynamically significant CAD.

## 1. Introduction

The Chinese population is suffering from an increase in cardiovascular diseases (CVD). There are approximately 330 million CVD patients in China, of whom 11.39 million have coronary artery disease (CAD). Notably, the mortality of CAD is still on the rise, especially in rural areas (1).

The mainstay of treatment for CAD is percutaneous coronary intervention (PCI). Patients with obstructive CAD receive PCI based on angiography, which remains the most widely used approach (2). However, coronary angiography (CAG) has the limitation of only evaluating the anatomy of lesions, but not their relationship with myocardial ischemia objectively and accurately (3). The most extensively used approach for assessing coronary physiology is fractional flow reserve (FFR), which can accurately identify hemodynamically significant coronary stenosis by measuring coronary pressure during cardiac catheterization (4). In the presence or absence of hyperemia-inducing agents, physiological measurement based on pressure wire is more accurate at detecting hemodynamically significant CAD than angiography alone (3, 5, 6). Several randomized trials have demonstrated that physiology-guided revascularization strategy based on pressure wire improves clinical outcomes by identifying hemodynamically significant CAD (7–11). FFR is currently recommended to guide PCI in stable CAD patients with 50%–90% visual stenosis on the CAG (12, 13). Quantitative flow ratio (QFR) is a recently developed approach for physiologically evaluating coronary stenosis by computing FFR based on three-dimensional angiographic reconstruction and hydrodynamic algorithms without the use of pressure wires and hyperemia-inducing agents. The FAVOR (Functional Diagnostic Accuracy of Quantitative Flow Ratio in Online Assessment of Coronary Stenosis) II China study demonstrated that QFR had excellent diagnostic accuracy at both patient- (92.4%) and vessel-level (92.7%) with invasive FFR as reference (14). Moreover, the FAVOR III China study concluded that the revascularization strategy guided by QFR improved 1- and 2-year clinical outcomes and led to fewer stents and contrast agents, less radiation exposure and shorter procedure time compared with classic angiography-based guidance (15, 16). Identification of hemodynamically significant CAD is crucial to improving clinical outcomes and reducing burden. The application of CAG and PCI has boosted remarkably in China, but the CAG positive rate in patients with suspected CAD is low (17). Hence, it is imperative to seek dependable markers of hemodynamically significant CAD to improve early diagnosis and risk stratification.

Epicardial adipose tissue (EAT) is a reservoir of fat with unique physiological properties, positioned between the myocardium and the visceral layer of the pericardium and sharing the same blood supply as the myocardium (18). EAT is metabolically active, and abundant in proatherogenic, proinflammatory, and prothrombotic adipocytokines (19). EAT can be evaluated with imaging techniques, mainly including echocardiography, cardiac CT and cardiac MRI. Echocardiography can be used to measure EAT thickness, with the advantages of low cost and accessibility and the limitations of acoustic window and operator dependence. Cardiac CT and cardiac MRI can provide a comprehensive assessment of EAT due to their high spatial resolution, such as location-specific EAT thickness and EAT volume (18). Ample evidence reveals that EAT correlates directly with the development and progression of CAD (20, 21). EAT thickness, especially left atrioventricular groove (AVG) EAT thickness, is significantly associated with obstructive CAD (22, 23). EAT volume is proportional to atherosclerosis, coronary artery calcium score (CACs), the risk of CAD, the presence of obstructive and vulnerable plaques (20, 24–27), as well as the occurrence of adverse clinical outcomes (28). However, the majority of studies were carried out in the western population and placed emphasis on the association between EAT and obstructive CAD. Additional investigations are needed to study the connection between EAT and hemodynamically significant CAD to improve risk stratification and clinical outcomes. Herein, we measured EAT volume based on coronary computed tomography angiography (CCTA) images and assessed the presence and severity of hemodynamically significant CAD by QFR to investigate the impact of EAT volume on hemodynamically significant CAD in Chinese patients with known or suspected CAD and attempt to provide a new theoretical basis for CAD severity prediction.

## 2. Materials and methods

### 2.1. Study population

**Figure 1:**
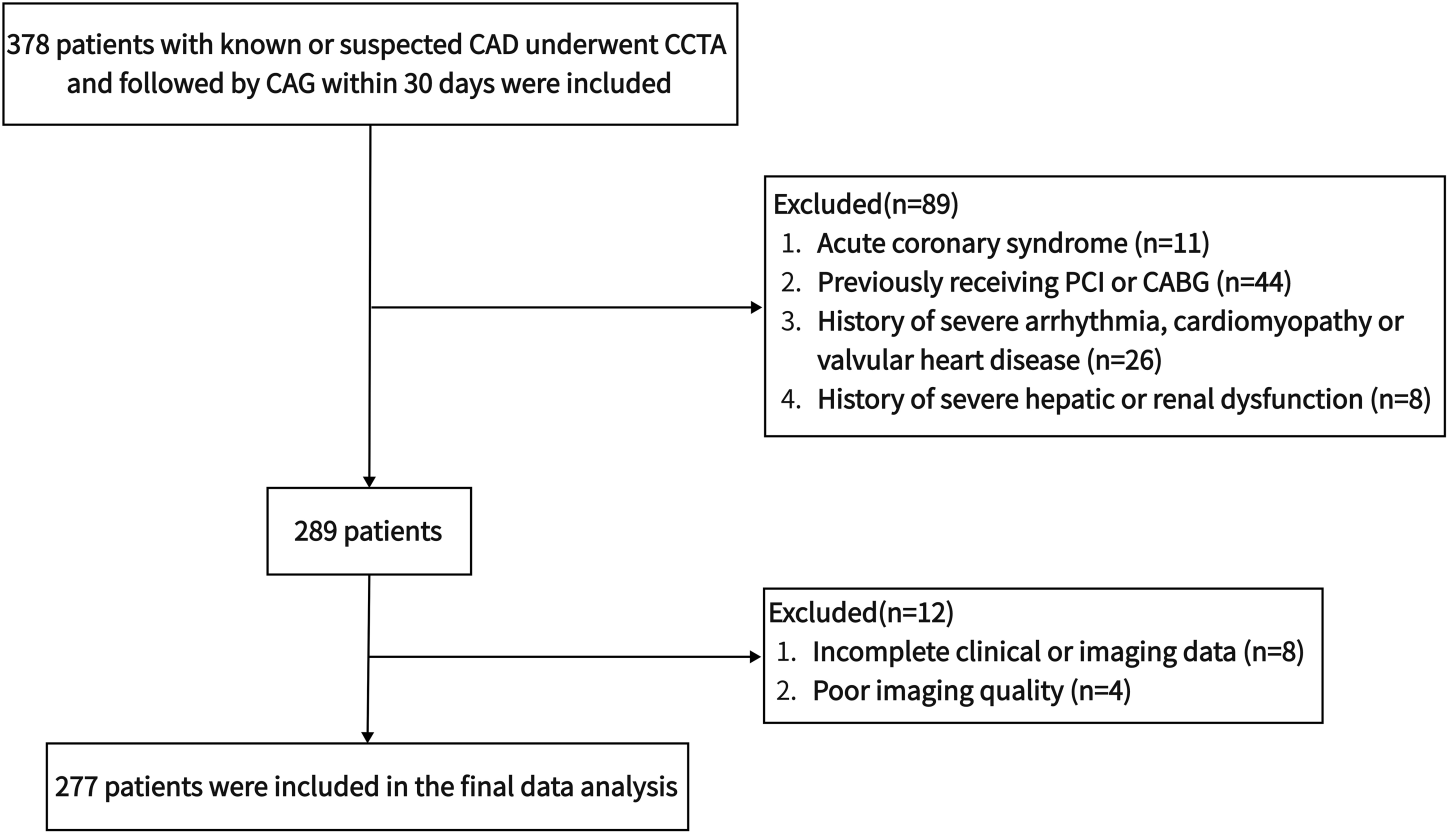
The flowchart of population selection. CABG, coronary artery bypass grafting; CAD, coronary artery disease; CAG, coronary angiography; CCTA, coronary computed tomography angiography; PCI, percutaneous coronary intervention.

We retrospectively enlisted 378 consecutive patients who underwent CAG and CCTA to assess known or suspected CAD at the Affiliated Hangzhou First People's Hospital, Zhejiang University School of Medicine from December 2019 to January 2021. All subjects received CAG within 30 days after CCTA. Hypertension was defined as using antihypertensive drugs or at least two blood pressure records ≥140/90 mmHg. The determination of diabetes mellitus was based on taking hypoglycemic medications, glycated hemoglobin (HbA1c) ≥6.5% or fasting blood glucose (FBG) ≥7.0 mmol/L (29). Receiving lipid-lowering medications, total cholesterol (TC) ≥6.2 mmol/L, triglycerides ≥2.3 mmol/L, low-density lipoprotein (LDL) ≥4.1 mmol/L or high-density lipoprotein (HDL) ≤1.0 mmol/L was considered diagnostic criteria for dyslipidemia (30). Smoking within the past year was regarded as active smoking. Population characteristics were extracted from patients’ electronic medical records. Body surface area and body mass index (BMI) were computed using height and weight. Exclusion criteria were: acute coronary syndrome, previously receiving PCI or coronary artery bypass grafting, history of severe arrhythmia, cardiomyopathy or valvular heart disease, history of severe hepatic or renal dysfunction, incomplete clinical or imaging data, poor imaging quality, age <18 years or pregnancy. The final data analysis included 277 patients. Figure 1 illustrates the population selection flowchart. The Medical Ethics Committee of the Affiliated Hangzhou First People's Hospital, Zhejiang University School of Medicine authorized the protocol and waiver of informed consent for this retrospective observational study.

### 2.2. CCTA examination

The second-generation dual-source CT system with 128 slices (SOMATOM Flash, Siemens) was used for image acquisition. The scanning protocol consisted of two parts: non-contrast-enhanced and contrast-enhanced cardiac CT imaging. For the non-contrast-enhanced imaging, the following scan settings were employed: tube voltage, 120 kV; tube current, 75 mAs; slice thickness, 3 mm; increment, 1.5 mm. For the contrast-enhanced imaging, a double-syringe injector was used to intravenously inject 65 ml contrast medium and 30 ml saline at a rate of 5 ml/s, and the injection was triggered by a threshold of 100 hounsfield unit (HU). All scanning was performed using a step-and-shot approach at a step-by-step distance of 38.4 mm. All imaging was conducted using a prospectively gated electrocardiogram-triggered sequential protocol with image acquisition triggered at 30%–80% of the R-R interval. The scan settings were as follows: rotation time, 0.25–0.28 s; tube voltage, 100 kV; tube current, 350–650 mAs; slice thickness, 0.75 mm; increment, 0.5 mm. CCTA was acquired after the intravenous injection of 370 mg I/ml iopromide (Ultravist, Bayer). The algorithms for optimization of electrocardiogram-gated reconstruction were applied to reconstruct horizontal axial images.

### 2.3. EAT volume and CACs measurement

The CCTA images were all post-processed by specialized radiologists on an offline workstation (Syngo.Via, Siemens). An experienced observer who turned a blind eye to the patient's medical history measured EAT volume and CACs. The adipose tissue contained in the pericardium with attenuation ranges between −190 and −30 HU was considered as EAT (31) and quantified by manually outlining the pericardium from the pulmonary trunk to the cardiac apex in axial slices of 0.75 mm thickness (32). EAT volume was automatically calculated by multiplying the sum of EAT cross-sectional area of each slice and the slice thickness. The semi-automatic software (Syngo.Via Volume, Siemens) was used throughout the full measuring procedure. The intraclass correlation coefficients for intra- and inter-observer reproducibility for measuring EAT volume were 0.982 [95% confidence interval (CI), 0.961–0.992] and 0.975 (95% CI, 0.881–0.992). Coronary artery calcification (CAC), defined as the area with attenuation values above 130 HU in the coronary artery, was evaluated by the Agatston coronary calcification score (33). CACs was calculated by adding the calcium score of each coronary artery.

### 2.4. CAG and QFR measurement

CAG was completed by experienced interventional cardiologists according to standard practice. Visual assessment of each coronary segment of major coronary arteries (such as left main coronary artery, left anterior descending coronary artery, left circumflex coronary artery and right coronary artery) was performed in at least two coronary angiographic images with different projection angles. ≥50% diameter stenosis (DS) in the left main coronary artery or ≥70% DS in at least one of the remaining major coronary arteries and their main branches was used to determine obstructive CAD (34). DS_max_ was defined as the maximum DS in all major coronary arteries.

All coronary angiographic images were transferred to the AngioPlus system (Pulse Medical Imaging Technology, Shanghai, China) for QFR assessment. Measurement of QFR was performed by an experienced operator who was oblivious to the patients’ medical history in all major coronary arteries. Based on the standard operating procedure as extensively described previously (15, 16), the QFR value for each location of the target vessel was automatically shown on the pullback curve by the AngioPlus system. According to invasive FFR, the diagnostic accuracy of QFR was excellent at both the patient- and vessel-level (14). The intraclass correlation coefficients for intra- and inter-observer reproducibility for measuring QFR were 0.991 (95% CI, 0.985–0.995) and 0.983 (95% CI, 0.973–0.989). QFR_min_ was defined as the minimum QFR in all major coronary arteries. Hemodynamically significant CAD was defined as QFR_min_ ≤0.80.

**Figure 2:**
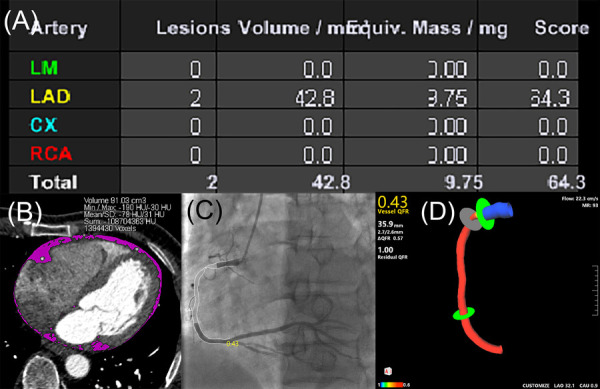
Case example of CACs calculation, EAT volume quantification and QFR assessment. (**A**) CACs was calculated by the sum of calcium score of each coronary artery according to the Agatston coronary calcification score. (**B**) EAT volume was quantified by manually tracing pericardium (pink). (**C**) QFR assessment in two-dimensional mode derived from CAG (QFR value of 0.43 in the RCA). (**D**) QFR assessment in three-dimensional mode derived from CAG (QFR value of 0.43 in the RCA). CACs, coronary artery calcium score; CAG, coronary angiography; CX, left circumflex coronary artery; EAT, epicardial adipose tissue; LAD, left anterior descending coronary artery; LM, left main coronary artery; QFR, quantitative flow ratio; RCA, right coronary artery.

The measurements of CACs, EAT volume and QFR were shown in Figure 2.

### 2.5. Statistical analysis

When continuous variables were normally distributed or not, they were presented as the mean ± standard deviation (SD) or median (25th percentile, 75th percentile), respectively. Student's *t*-test for normal distributions or Mann-Whitney *U* test for skewed distributions was applied to compare continuous variables from two separate data sets. ANOVA or Kruskal-Wallis test was adopted to test the distribution differences of continuous variables among groups, and *post-hoc* tests were performed with Bonferroni corrections (adjusted *P*-value <0.05 for significance on ANOVA or Kruskal-Wallis test). Categorical variables were described as absolute numbers and percentages, and the chi-square test or Fisher exact test was employed to analyze them. We measured the reproducibility of QFR and EAT volume with the intraclass correlation coefficients in a random sample of 25 patients. The correlation coefficient *R* or Spearman's rho was used to evaluate the association between two variables. Univariate and multivariate logistic regression analysis was implemented to evaluate the relationship between EAT volume and hemodynamically significant CAD. The connection between EAT volume and QFR_min_ was evaluated through univariate and multivariate linear regression analysis. For multivariate analysis, only variables with *P*-value <0.1 in univariate analysis were taken into account. We excluded multi-collinearity between selected variables based on variance inflation factor >5. The diagnostic efficacy for identifying the existence of hemodynamically significant CAD was assessed using receiver operating characteristics (ROC) curve analysis. According to the DeLong approach, the evaluation of discriminatory power was carried out by measuring and comparing the area under the ROC curve (AUC) (35). The optimal cutoff values and diagnostic performance features [such as sensitivity, specificity, positive predictive value (PPV) and negative predictive value (NPV)] were obtained by applying the Youden index in ROC curve analysis.

Data were analyzed with SPSS Statistics version 26.0 (IBM Corp, Armonk, New York, United States) and MedCalc version 19.8 (MedCalc Software Ltd, Ostend, Belgium). All tests were performed two-sided, and statistical significance was defined as a *P*-value <0.05.

## 3. Results

### 3.1. Study population

Table 1 displayed the demographic and clinical characteristics of patients with or without hemodynamically significant CAD. A total of 277 subjects were included in this study, of whom 112 were diagnosed with hemodynamically significant CAD and 143 with obstructive CAD. We found that 76.9% of obstructive CAD patients were complicated with hemodynamically significant CAD, which indicated that although obstructive CAD was highly associated with myocardial ischemia, there were still some obstructive CAD that failed to induce myocardial ischemia. This finding was consistent with the FAME (Fractional Flow Reserve Versus Angiography in Multivessel Evaluation) study (36). Compared with patients without hemodynamically significant CAD, patients with hemodynamically significant CAD were more frequently male (62.5%), had higher age, BMI and body surface area, along with a higher prevalence of active smoking (34.8%), hypertension (81.3%), diabetes mellitus (41.1%) and dyslipidemia (69.6%). Exception for HDL, triglycerides, LDL, creatinine, uric acid, FBG, HbA1c, DS_max_ and CACs were also higher in patients with hemodynamically significant CAD. In addition, patients with hemodynamically significant CAD displayed more increased EAT volume than patients without hemodynamically significant CAD (135.56 ± 38.23 cm^3^ vs. 103.98 ± 35.93 cm^3^; *P *< 0.001). After adjusting for BMI (5.48 ± 1.33 vs. 4.35 ± 1.34; *P *< 0.001) and body surface area (58.13 ± 15.18 vs. 45.40 ± 14.99; *P *< 0.001), the between-group difference remained significant.

**Table 1:** Population characteristics.

Variable	Group		*P*-value
	Hemodynamically significant CAD (*N* = 112)	No hemodynamically significant CAD (*N* = 165)	
Demographics
Age, years	66.74 ± 10.16	63.61 ± 9.97	**0** **.** **011**
Men, *n* (%)	70 (62.5)	78 (47.3)	**0** **.** **013**
BMI, kg/m^2^	24.63 ± 3.03	23.76 ± 3.11	**0** **.** **021**
Body surface area, m^2^	2.32 ± 0.19	2.28 ± 0.16	**0** **.** **045**
Clinical evaluation
Active smoking, *n* (%)	39 (34.8)	36 (21.8)	**0** **.** **017**
Hypertension, *n* (%)	91 (81.3)	111 (67.3)	**0** **.** **010**
Diabetes mellitus, *n* (%)	46 (41.1)	44 (26.7)	**0** **.** **012**
Dyslipidemia, *n* (%)	78 (69.6)	90 (54.5)	**0** **.** **012**
Obstructive CAD, *n* (%)	110 (98.2)	33 (20.0)	**<0** **.** **001**
Blood test
TC, mmol/L	4.41 ± 1.17	4.23 ± 1.03	0.163
Triglycerides, mmol/L	1.57 (1.19, 2.02)	1.24 (0.92, 1.94)	**0** **.** **003**
HDL, mmol/L	1.09 ± 0.26	1.19 ± 0.31	**0** **.** **005**
LDL, mmol/L	2.55 ± 0.96	2.33 ± 0.83	**0** **.** **041**
Creatinine, μmol/L	88.68 ± 15.44	84.66 ± 12.92	**0** **.** **020**
Uric acid, μmol/L	352.55 ± 92.18	328.99 ± 95.55	**0** **.** **042**
FBG, mmol/L	5.20 (4.60, 6.07)	4.90 (4.36, 5.52)	**0** **.** **006**
HbA1c, %	5.9 (5.5, 6.7)	5.6 (5.4, 6.1)	**0** **.** **002**
Imaging test
EF, %	64.0 (60.0, 68.0)	65.0 (61.0, 68.0)	0.269
DS_­max_, %	90.0 (85.0, 95.0)	50.0 (20.0, 60.0)	**<0** **.** **001**
QFR_min_	0.66 (0.27, 0.73)	0.95 (0.91, 0.97)	**<0** **.** **001**
Vessels with QFR ≤0.8	1 (1, 2)	0 (0, 0)	**<0** **.** **001**
CACs	190.75 (62.28, 577.75)	32.70 (0, 244.95)	**<0** **.** **001**
EAT volume, cm^3^	135.56 ± 38.23	103.98 ± 35.93	**<0** **.** **001**
EAT index1	5.48 ± 1.33	4.35 ± 1.34	**<0** **.** **001**
EAT index2	58.13 ± 15.18	45.40 ± 14.99	**<0** **.** **001**

Values are mean ± standard deviation, *n* (%), or median (25th percentile, 75th percentile). BMI, body mass index; CACs, coronary artery calcium score; CAD, coronary artery disease; DS, diameter stenosis; EAT, epicardial adipose tissue; EF, ejection fraction; FBG, fasting blood glucose; HbA1c, glycated hemoglobin; HDL, high-density lipoprotein; LDL, low-density lipoprotein; QFR, quantitative flow ratio; TC, total cholesterol. EAT index1, EAT volume adjusted for BMI (EAT volume/BMI); EAT index2, EAT volume adjusted for body surface area (EAT volume/body surface area).

Bold value is statistically significant.

Patients were stratified by tertiles of EAT volume to study the factors that influence EAT volume (Table 2). Mean EAT volume was 74.39 ± 15.77 cm^3^ (range, 21.57–93.44 cm^3^) in the bottom tertile group, 112.96 ± 12.14 cm^3^ (range, 93.66–133.71 cm^3^) in the middle tertile group and 162.94 ± 21.36 cm^3^ (range, 134.87–253.94 cm^3^) in the top tertile group, respectively. Compared to the bottom and middle tertile groups, the top tertile group had significantly higher BMI, body surface area, FBG, DS_max_ and CACs. Notably, the top tertile group showed the most vessels with QFR ≤0.8, whereas the bottom tertile group displayed the highest level of QFR_min_ [0.94; interquartile range (IQR), 0.83–0.97; *P *< 0.001]. There was an increase in hemodynamically significant CAD mostly in the top tertile group (60, 65.2%; *P *< 0.001), which did not differ between the bottom (20, 21.7%) and middle tertile groups (32, 34.4%).

**Table 2:** Population characteristics stratified by tertiles of EAT volume.

Variable	Bottom tertile (*N* = 92)	Middle tertile (*N* = 93)	Top tertile (*N* = 92)	*P*-value
EAT volume
EAT, minium-maxium, cm^3^	21.57–93.44	93.66–133.71	134.87–253.94	
EAT, mean ± SD, cm^3^	74.39 ± 15.77	112.96 ± 12.14	162.94 ± 21.36	
EAT, median (P25, P75), cm^3^	78.96 (63.03, 87.98)	112.70 (102.15, 122.86)	160.21 (145.36, 175.47)	
Demographics
Age, years	62.64 ± 9.43	65.37 ± 9.72	66.61 ± 10.94*	**0.025**
Men, *n* (%)	41 (44.6)	53 (57.0)	54 (58.7)	0.111
BMI, kg/m^2^	22.29 ± 2.79	24.22 ± 2.62*	25.82 ± 2.87*^,^**	**<0** **.** **001**
Body surface area, m^2^	2.20 ± 0.15	2.31 ± 0.13*	2.38 ± 0.18*^,^**	**<0** **.** **001**
Blood test
TC, mmol/L	4.32 ± 1.09	4.28 ± 1.09	4.31 ± 1.10	0.974
Triglycerides, mmol/L	1.15 (0.88, 1.58)	1.42 (1.05, 2.01)*	1.63 (1.15, 2.23)*	**<0** **.** **001**
HDL, mmol/L	1.26 ± 0.30	1.10 ± 0.27*	1.08 ± 0.28*	**<0** **.** **001**
LDL, mmol/L	2.37 ± 0.87	2.44 ± 0.93	2.44 ± 0.87	0.825
Creatinine, μmol/L	81.21 ± 11.38	87.95 ± 14.57*	89.68 ± 14.79*	**<0** **.** **001**
Uric acid, μmol/L	307.02 ± 80.42	349.52 ± 95.31*	358.89 ± 100.14*	**<0** **.** **001**
FBG, mmol/L	4.75 (4.22, 5.26)	4.97 (4.39, 5.72)	5.28 (4.67, 6.43)*^,^**	**<0** **.** **001**
HbA1c, %	5.5 (5.2, 5.9)	5.7 (5.5, 6.5)*	6.0 (5.6, 7.0)*	**<0** **.** **001**
Imaging test
EF, %	64.5 (61.0, 69.0)	64.0 (61.0, 68.0)	65.0 (60.3, 68.0)	0.553
DS_max_, %	60.0 (30.0, 80.0)	60.0 (30.0, 85.0)	80.0 (50.0, 90.0)*,**	**<0** **.** **001**
QFR_min_	0.94 (0.83, 0.97)	0.89 (0.73, 0.96)	0.74 (0.53, 0.92)*^,^**	**<0** **.** **001**
Vessels with QFR ≤0.8	0 (0, 0)	0 (0, 1)	1 (0, 1)*^,^**	**<0** **.** **001**
CACs	48.45 (0, 281.95)	40.80 (0.35, 298.95)	171.85 (40.35, 539.25)*^,^**	**0** **.** **002**
Clinical evaluation
Active smoking, *n* (%)	23 (25.0)	23 (24.7)	29 (31.5)	0.501
Hypertension, *n* (%)	54 (58.7)	73 (78.5)*	75 (81.5)*	**0** **.** **001**
Diabetes mellitus, *n* (%)	15 (16.3)	32 (34.4)*	43 (46.7)*	**<0** **.** **001**
Dyslipidemia, *n* (%)	42 (45.7)	60 (64.5)*	66 (71.7)*	**0** **.** **001**
Obstructive CAD, *n* (%)	36 (39.1)	46 (49.5)	61 (66.3)*	**0** **.** **001**
Hemodynamically significant CAD, *n* (%)	20 (21.7)	32 (34.4)	60 (65.2)*^,^**	**<0** **.** **001**

Values are mean ± standard deviation, *n* (%), or median (25th percentile, 75th percentile). BMI, body mass index; CACs, coronary artery calcium score; CAD, coronary artery disease; DS, diameter stenosis; EAT, epicardial adipose tissue; EF, ejection fraction; FBG, fasting blood glucose; HbA1c, glycated hemoglobin; HDL, high-density lipoprotein; LDL, low-density lipoprotein; QFR, quantitative flow ratio; TC, total cholesterol.

Bold value is statistically significant.

* *P *< 0.05 vs. bottom tertile.

** *P *< 0.05 vs. middle tertile.

### 3.2. Association of EAT volume with demographic characteristics, laboratory and imaging values

**Figure 3:**
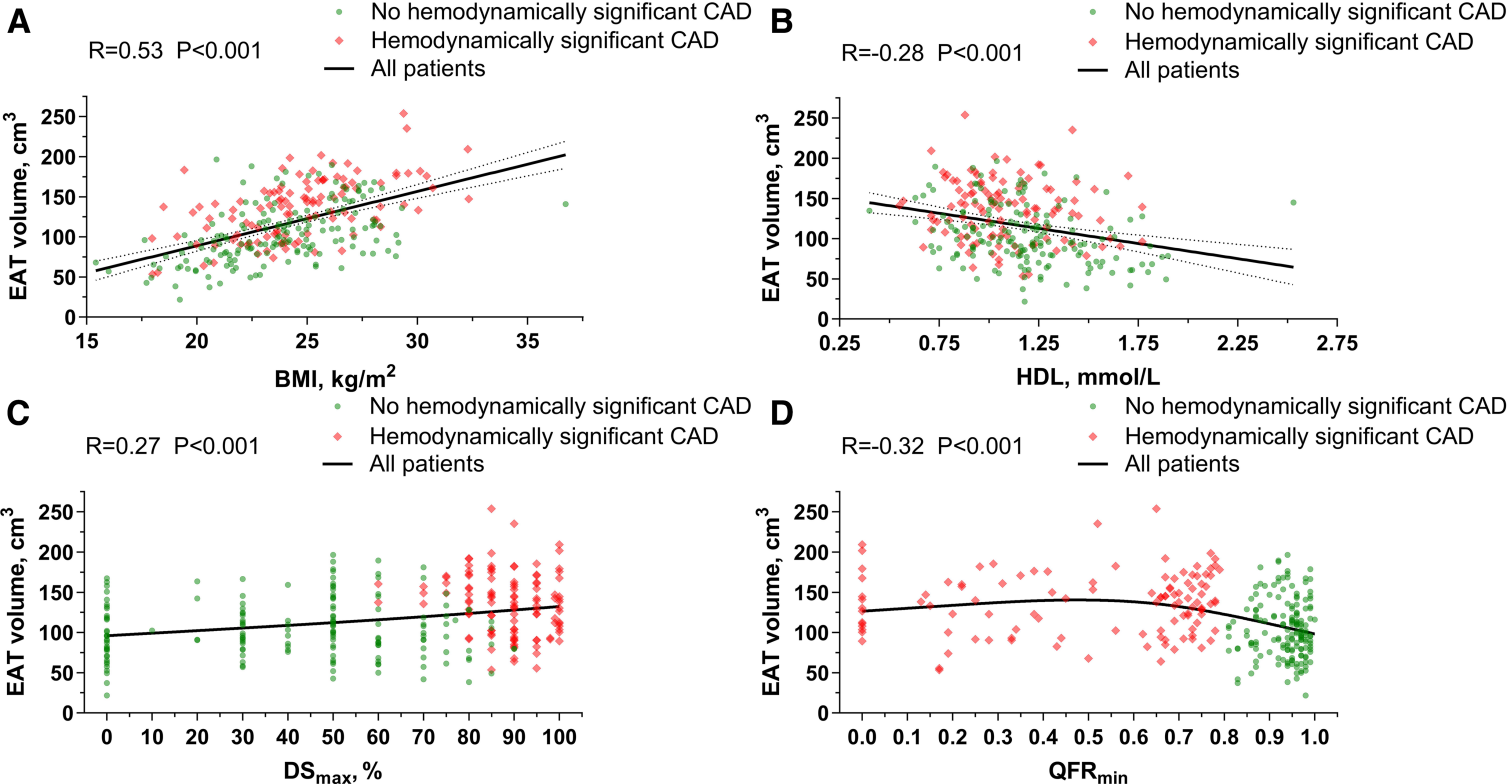
Association of EAT volume with BMI, HDL, dS_max_ and QFR_min_. (**A**) Correlation between EAT volume and BMI. (**B**) Correlation between EAT volume and HDL. (**C**) Correlation between EAT volume and DS_max_. (**D**) Correlation between EAT volume and QFR_min_. BMI, body mass index; CAD, coronary artery disease; DS, diameter stenosis; EAT, epicardial adipose tissue; HDL, high-density lipoprotein; QFR, quantitative flow ratio.

In the general population, EAT volume was directly correlated with age (*R*, 0.17; *P* = 0.004), BMI (*R*, 0.53; *P *< 0.001), body surface area (*R*, 0.42; *P *< 0.001), triglycerides (*R*, 0.28; *P *< 0.001), creatinine (*R*, 0.22; *P *< 0.001), uric acid (*R*, 0.27; *P *< 0.001), FBG (*R*, 0.27; *P *< 0.001), HbA1c (*R*, 0.33; *P *< 0.001), CACs (*R*, 0.18; *P* = 0.003), DS_max_ (*R*, 0.27; *P *< 0.001) and the number of vessels with QFR ≤0.8 (*R*, 0.34; *P *< 0.001), but inversely associated with HDL (*R*, −0.28; *P *< 0.001) and QFR_min_ (*R*, −0.32; *P *< 0.001) (Figure 3 and Supplementary Figures S1, S2).

### 3.3. Association of hemodynamically significant CAD with demographic characteristics, cardiometabolic disease, laboratory and imaging values

**Figure 4:**
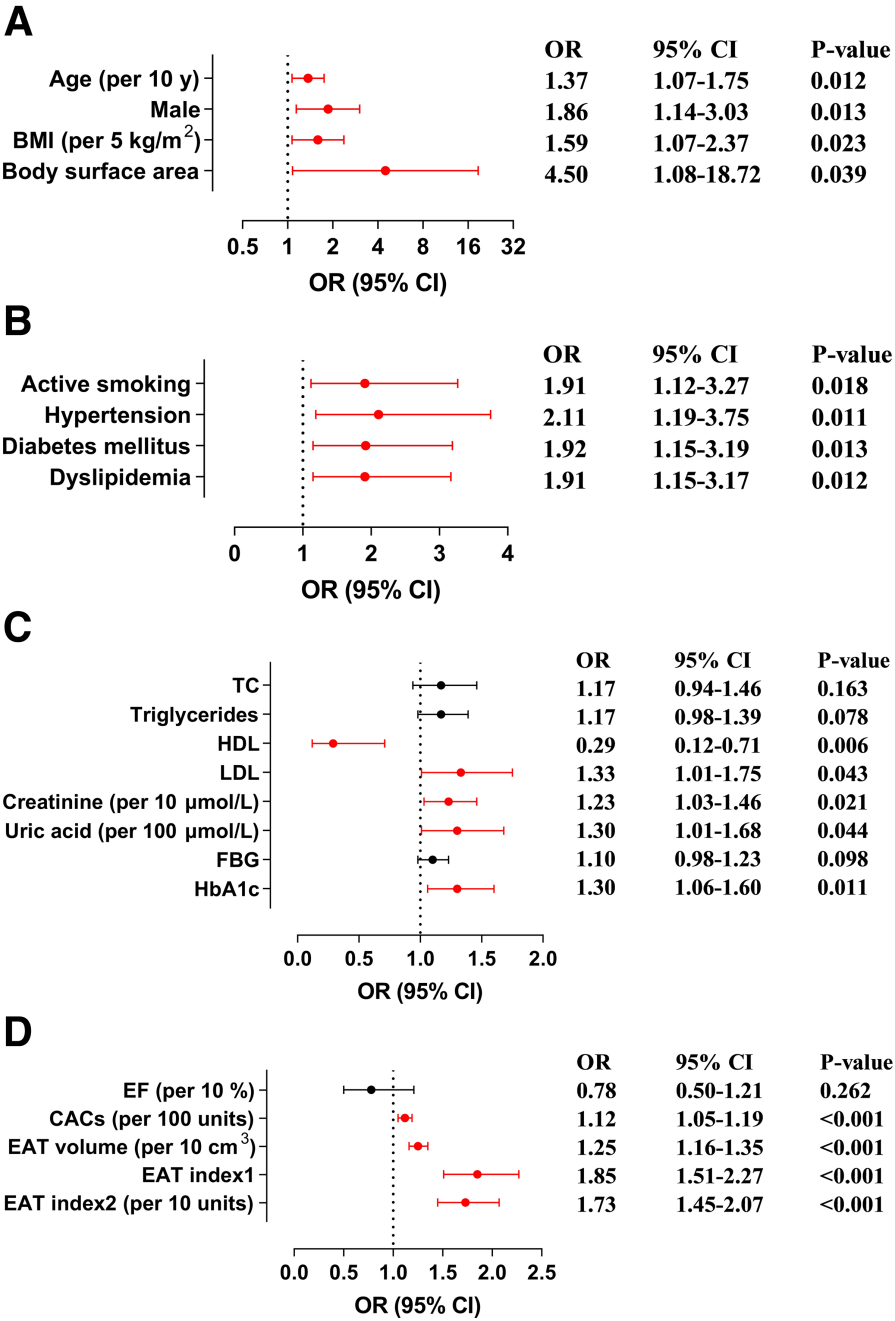
Association of hemodynamically significant CAD with demographic characteristics, cardiometabolic disease, laboratory and imaging values. (**A**) Forest plots illustrate the association between hemodynamically significant CAD and demographic characteristics. (**B**) Forest plots illustrate the association between hemodynamically significant CAD and cardiometabolic disease. (**C**) Forest plots illustrate the association between hemodynamically significant CAD and laboratory values. (**D**) Forest plots illustrate the association between hemodynamically significant CAD and imaging values. BMI, body mass index; CACs, coronary artery calcium score; CAD, coronary artery disease; CI, confidence interval; EAT, epicardial adipose tissue; EF, ejection fraction; FBG, fasting blood glucose; HbA1c, glycated hemoglobin; HDL, high-density lipoprotein; LDL, low-density lipoprotein; OR, odds ratio; TC, total cholesterol.

With univariate logistic regression analysis, we assessed the relationship between hemodynamically significant CAD and demographic characteristics, cardiometabolic disease, laboratory and imaging values (Figure 4). Herein, hemodynamically significant CAD had a significant positive association with age [per 10 years; odds ratio (OR), 1.37; 95% CI, 1.07–1.75; *P* = 0.012], male (OR, 1.86; 95% CI, 1.14–3.03; *P* = 0.013), BMI (per 5 kg/m^2^; OR, 1.59; 95% CI, 1.07–2.37; *P* = 0.023), body surface area (OR, 4.50; 95% CI, 1.08–18.72; *P* = 0.039), active smoking (OR, 1.91; 95% CI, 1.12–3.27; *P* = 0.018), hypertension (OR, 2.11; 95% CI, 1.19–3.75; *P* = 0.011), diabetes mellitus (OR, 1.92; 95% CI, 1.15–3.19; *P* = 0.013), dyslipidemia (OR, 1.91; 95% CI, 1.15–3.17; *P* = 0.012), LDL (OR, 1.33; 95% CI, 1.01–1.75; *P* = 0.043), creatinine (per 10 μmol/L; OR, 1.23; 95% CI, 1.03–1.46; *P* = 0.021), uric acid (per 100 μmol/L; OR, 1.30; 95% CI, 1.01–1.68; *P* = 0.044), HbA1c (OR, 1.30; 95% CI, 1.06–1.60; *P* = 0.011), and CACs (per 100 units; OR, 1.12; 95% CI, 1.05–1.19; *P *< 0.001), but a strong negative relationship with HDL (OR, 0.29; 95% CI, 0.12–0.71; *P* = 0.006). EAT volume and hemodynamically significant CAD were also positively correlated (per 10 cm^3^; OR, 1.25; 95% CI, 1.16–1.35; *P *< 0.001), which was maintained after adjustment for BMI (OR, 1.85; 95% CI, 1.51–2.27; *P *< 0.001) and body surface area (per 10 units; OR, 1.73; 95% CI, 1.45–2.07; *P *< 0.001).

### 3.4. Multivariate logistic regression analysis between EAT volume and hemodynamically significant CAD

There was also a significant positive correlation between EAT volume and hemodynamically significant CAD in multivariate logistic regression analysis (Table 3). In multivariate-adjusted model 1 (adjusted for age and sex), a per-SD increase in EAT volume had an OR of 2.34 for hemodynamically significant CAD (95% CI, 1.74–3.14; *P *< 0.001), and the OR increased with EAT volume tertile. In multivariate-adjusted model 2 (adjusted for age, sex, BMI, body surface area, active smoking, hypertension, diabetes mellitus, dyslipidemia, creatinine, uric acid and CACs), we confirmed EAT volume was independently and positively correlated with hemodynamically significant CAD (per SD cm^3^; OR, 2.78; 95% CI, 1.86–4.15; *P *< 0.001). Moreover, with the bottom tertile of EAT volume as control, the OR for the top tertile of EAT volume was 6.71 (95% CI, 2.80–16.09; *P *< 0.001), while the OR for the middle tertile of EAT volume was not statistically significant. Multivariate-adjusted model 3 (adjusted for age, sex, BMI, body surface area, active smoking, hypertension, triglycerides, HDL, LDL, FBG, HbA1c, creatinine, uric acid and CACs) yielded similar results.

**Table 3:** Multivariate logistic regression analysis for effect of EAT volume on hemodynamically significant CAD.

Variable	Multivariate-adjusted		Multivariate-adjusted		Multivariate-adjusted	
	Model 1		Model 2		Model 3	
	OR (95% CI)	*P*-value	OR (95% CI)	*P*-value	OR (95% CI)	*P*-value
EAT volume
EAT (per SD)	2.34 (1.74–3.14)	**<0** **.** **001**	2.78 (1.86–4.15)	**<0** **.** **001**	2.79 (1.87–4.17)	**<0** **.** **001**
EAT (per tertile)	2.49 (1.77–3.49)	**<0** **.** **001**	2.71 (1.75–4.19)	**<0** **.** **001**	2.74 (1.76–4.29)	**<0** **.** **001**
Tertiles
Bottom tertile^a^	1		1		1	
Middle tertile	1.65 (0.84–3.22)	0.145	1.77 (0.82–3.83)	0.149	1.62 (0.73–3.57)	0.235
Top tertile	5.86 (3.00–11.43)	**<0** **.** **001**	6.71 (2.80–16.09)	**<0** **.** **001**	6.80 (2.78–16.63)	**<0** **.** **001**

Multivariate-adjusted model 1 was adjusted for age and sex; multivariate-adjusted model 2 was adjusted for age, sex, body mass index, body surface area, active smoking, hypertension, diabetes mellitus, dyslipidemia, creatinine, uric acid and coronary artery calcium score; multivariate-adjusted model 3 was adjusted for age, sex, body mass index, body surface area, active smoking, hypertension, triglycerides, high-density lipoprotein, low-density lipoprotein, fasting blood glucose, glycated hemoglobin, creatinine, uric acid and coronary artery calcium score. CAD, coronary artery disease; CI, confidence interval; EAT, epicardial adipose tissue; OR, odds ratio; SD, standard deviation.

Bold value is statistically significant.

^a^ As control.

### 3.5. Univariate and multivariate linear regression analysis between EAT volume and QFR_min_

EAT volume and QFR_min_ were analyzed linearly to study the relationship between EAT volume and hemodynamically significant CAD severity (Table 4). According to the univariate analysis, EAT volume and QFR_min_ were negatively associated (per SD cm^3^; *β* coefficient, −0.073; 95% CI, −0.105 to −0.041; *P *< 0.001). Based on the multivariate analysis, QFR_min_ was independently correlated with HDL (*β* coefficient, 0.190; 95% CI, 0.069 to 0.311; *P* = 0.002), LDL (*β* coefficient, −0.051; 95% CI, −0.088 to −0.014; *P* = 0.007), CACs (per 100 units; *β* coefficient, −0.011; 95% CI, −0.018 to −0.005; *P* = 0.001) and EAT volume (per SD cm^3^; *β* coefficient, −0.066; 95% CI, −0.102 to −0.030; *P *< 0.001). Moreover, the multivariate-adjusted model (adjusted for CACs and traditional risk factors: age, sex, BMI, body surface area, active smoking, hypertension, diabetes mellitus, dyslipidemia, creatinine and uric acid) yielded a similar association between EAT volume and QFR_min_ (per SD cm^3^; *β* coefficient, −0.068; 95% CI, −0.109 to −0.027; *P* = 0.001).

**Table 4:** Univariate and multivariate linear regression analysis for effect of EAT volume on QFR_min_.

Variable	Univariate analysis		Multivariate analysis	
	*β* coefficient (95% CI)	*P*-value	*β* coefficient (95% CI)	*P*-value
Age	−0.02 (−0.005, 0.001)	0.241		
Male	−0.097 (−0.162, −0.033)	**0** **.** **003**	−0.084 (−0.178, 0.010)	0.080
BMI	−0.007 (−0.018, 0.003)	0.189		
Body surface area	−0.246 (−0.435, −0.057)	**0** **.** **011**	0.248 (−0.019, 0.515)	0.068
Active smoking	−0.136 (−0.208, −0.064)	**<0** **.** **001**	−0.071 (−0.151, 0.008)	0.078
Hypertension	−0.057 (−0.131, 0.016)	0.126		
Diabetes mellitus	−0.067 (−0.137, 0.003)	0.059	−0.016 (−0.085, 0.053)	0.653
Dyslipidemia	−0.099 (−0.165, −0.033)	**0** **.** **003**		
TC	−0.018 (−0.048, 0.012)	0.246		
Triglycerides	−0.021 (−0.041, 0)	**0** **.** **046**	0 (−0.020, 0.021)	0.966
HDL	0.213 (0.103, 0.322)	**<0** **.** **001**	0.190 (0.069, 0.311)	**0** **.** **002**
LDL	−0.037 (−0.074, −0.001)	**0** **.** **046**	−0.051 (−0.088, −0.014)	**0** **.** **007**
Creatinine	−0.002 (−0.005, 0)	**0** **.** **048**	0.001 (−0.002, 0.003)	0.676
Uric acid	0 (−0.001, 0)	0.094	0 (0, 0.001)	0.446
FBG	−0.006 (−0.022, 0.009)	0.412		
HbA1c	−0.018 (−0.044, 0.009)	0.193		
EF	0.006 (0, 0.012)	**0** **.** **034**	0.004 (−0.001, 0.010)	0.127
CACs (per 100)	−0.014 (−0.020, −0.007)	**<0** **.** **001**	−0.011 (−0.018, −0.005)	**0** **.** **001**
EAT volume (per SD)	−0.073 (−0.105, −0.041)	**<0** **.** **001**	−0.066 (−0.102, −0.030)	**<0** **.** **001**

BMI, body mass index; CACs, coronary artery calcium score; CI, confidence interval; EAT, epicardial adipose tissue; EF, ejection fraction; FBG, fasting blood glucose; HbA1c, glycated hemoglobin; HDL, high-density lipoprotein; LDL, low-density lipoprotein; QFR, quantitative flow ratio; SD, standard deviation; TC, total cholesterol.

Bold value is statistically significant.

### 3.6. Diagnostic performance of imaging indicators for hemodynamically significant CAD

**Figure 5:**
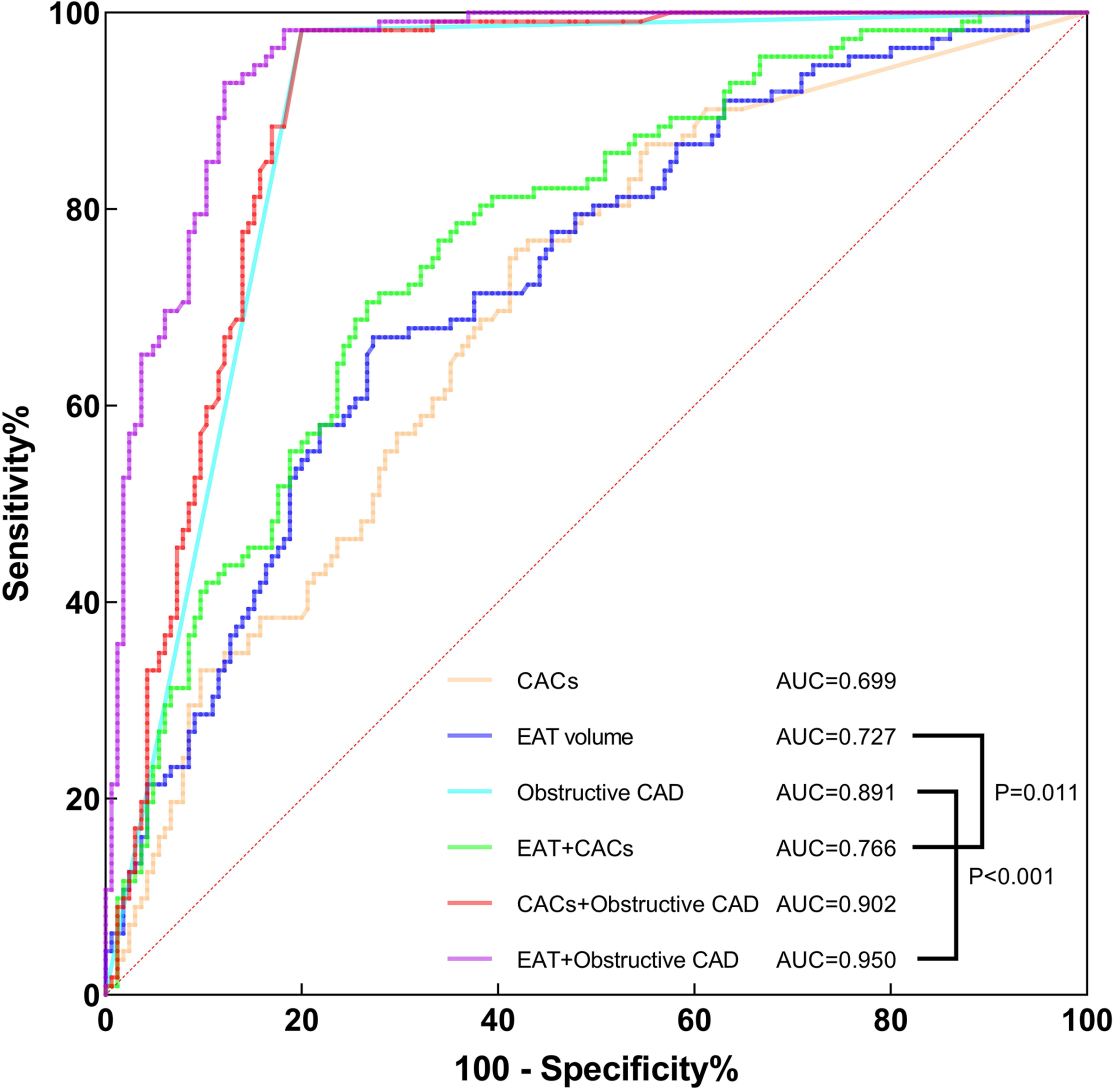
ROC curve analysis of CACs, EAT volume, obstructive CAD, combined EAT + CACs, combined CACs + obstructive CAD and combined EAT + obstructive CAD for identifying hemodynamically significant CAD. AUC, area under the curve; CACs, coronary artery calcium score; CAD, coronary artery disease; EAT, epicardial adipose tissue; ROC, receiver operating characteristics.

According to multivariate logistic regression analysis, CACs and EAT volume were identified as independent positive predictors for hemodynamically significant CAD. Obstructive CAD has been shown to be highly related to myocardial ischemia (36). Therefore, we conducted a ROC curve analysis of CACs, EAT volume, obstructive CAD and combinations to assess their diagnostic performance (Table 5 and Figure 5). In diagnosing hemodynamically significant CAD, obstructive CAD had a significantly higher AUC than CACs and EAT volume. When integrating EAT volume and obstructive CAD, we noticed that AUC for the combined method increased to 0.950, with a significant improvement over obstructive CAD (AUC, 0.950 vs. 0.891; *P *< 0.001). However, in combination with obstructive CAD, CACs failed to improve diagnostic performance significantly (AUC, 0.902 vs. 0.891; *P* = 0.336). In terms of noninvasive imaging indicators, EAT volume and CACs are of great interest. Both indicators had significantly higher AUCs combined than either indicator alone, with satisfactory sensitivity (70.5%), specificity (73.3%), PPV (64.2%) and NPV (78.6%).

**Table 5:** Diagnostic performance of imaging indicators for hemodynamically significant CAD.

Indicator	AUC (95% CI)	Best cutoffs	Youden index	Sensitivity, % (95% CI)	Specificity, % (95% CI)	PPV, % (95% CI)	NPV, % (95% CI)
CACs	0.699 (0.641–0.752)	>58.7	0.341	75.9 (66.9–83.5)	58.2 (50.3–65.8)	55.2 (50.0–60.3)	78.0 (71.4–83.5)
EAT volume, cm^3^	0.727 (0.670–0.779)	>120.74	0.397	67.0 (57.4–75.6)	72.7 (65.3–79.4)	62.5 (55.7–68.8)	76.4 (71.0–81.1)
Obstructive CAD	0.891 (0.848–0.925)		0.782	98.2 (93.7–99.8)	80.0 (73.1–85.8)	76.9 (71.0–81.9)	98.5 (94.3–99.6)
EAT + CACs	0.766 (0.712–0.815)	>0.389	0.439	70.5 (61.2–78.8)	73.3 (65.9–79.9)	64.2 (57.6–70.4)	78.6 (73.1–83.2)
CACs + Obstructive CAD	0.902 (0.861–0.935)	>0.025	0.782	98.2 (93.7–99.8)	80.0 (73.1–85.8)	76.9 (71.0–81.9)	98.5 (94.3–99.6)
EAT + Obstructive CAD	0.950 (0.917–0.973)	>0.526	0.807	92.9 (86.4–96.9)	87.9 (81.9–92.4)	83.9 (77.5–88.7)	94.8 (90.3–97.3)

AUC, area under curve; CACs, coronary artery calcium score; CAD, coronary artery disease; CI, confidence interval; EAT, epicardial adipose tissue; NPV, negative predictive value; PPV, positive predictive value.

## 4. Discussion

The findings of this study can be summarized as follows: (1) Patients with hemodynamically significant CAD displayed higher EAT volume than those without, and its prevalence increased across tertiles of EAT volume. (2) EAT volume correlated directly with age, BMI, triglycerides, creatinine, uric acid, FBG, HbA1c, CACs, DS_max_ and the number of vessels with QFR ≤0.8, but inversely with HDL and QFR_min_. (3) Multivariate logistic regression analysis indicated a significant and positive relationship between EAT volume and hemodynamically significant CAD independently of traditional factors and CACs. (4) According to multivariate linear regression analysis, EAT volume was independently and negatively associated with QFR_min_. (5) The diagnostic performance for hemodynamically significant CAD was substantially improved by adding EAT volume to obstructive CAD.

Due to its unobstructed connection with coronary arteries, EAT was first identified as a cause of coronary atherosclerosis in the early 2000s (18). Through the release of anti-inflammatory and pro-inflammatory cytokines, EAT acts as an endocrine gland that interacts with the coronary vessel wall and myocardium (37). Increases in EAT volume result in increased plaque composition burden and risk of obstructive CAD (26, 31, 38). Growing evidence has implicated EAT in the development and aggravation of CAD, making EAT an attractive marker for the primary prevention of CAD (25, 39). However, some studies failed to find similar positive correlation (40). In addition, the clinical implication of EAT in hemodynamically significant CAD is still largely unknown. Hence, we evaluated the relationship of EAT volume with hemodynamically significant CAD, and tried to determine the threshold of EAT volume that can best identify hemodynamically significant CAD. We found patients with hemodynamically significant CAD showed a higher level of EAT volume than in those without. After stratifying by tertiles of EAT volume, the prevalence of hemodynamically significant CAD increased across all groups. Based on the ROC curve analysis, the optimal cutoff value of EAT volume for predicting hemodynamically significant CAD was 120.74 cm^3^.

According to a prior study, EAT was significantly associated with metabolic syndrome risk factors (such as waist circumference, triglycerides, HDL, FBG and blood pressure) (41). A large meta-analysis of 41,534 individuals with cardiovascular risk showed that EAT volume was related to CAC and independently correlated with coronary artery stenosis and myocardial ischemia (42). Similarly, we found EAT volume correlated directly with age, BMI, triglycerides, creatinine, uric acid, FBG, HbA1c, CACs, DS_max_ and the number of vessels with QFR ≤0.8, but inversely with HDL and QFR_min_. Our results confirmed and extended the link between EAT and the risk factors for cardiovascular disease and metabolic syndrome. According to correlation analysis, EAT volume and BMI showed the highest correlation with a correlation coefficient R of 0.53. We further explored whether BMI could be used as a good predictor of hemodynamically significant CAD as EAT volume. Based on multivariate regression analysis, the association of BMI with hemodynamically significant CAD (OR, 1.05; 95% CI, 0.92–1.21; *P* = 0.466) and QFR_min_ (*β* coefficient, 0.001; 95% CI, −0.014 to 0.017; *P* = 0.880) was not statistically significant when adjusted for traditional risk factors and CACs. Thus, BMI could not be an independent predictor of hemodynamically significant CAD. In addition, EAT volume and creatinine displayed a statistically significant correlation (*R*, 0.22; *P *< 0.001), suggesting a possible association between renal insufficiency and EAT volume. Since patients with severe renal dysfunction were excluded from this study, subjects could only be divided into normal renal function group (creatinine ≤88.4 μmol/L; *N* = 175) and abnormal renal function group (creatinine >88.4 μmol/L; *N* = 102). We found that the abnormal renal function group showed a higher EAT volume than the normal renal function group (131.57 ± 36.73 cm^3^ vs. 108.11 ± 39.30 cm^3^; *P *< 0.001). The association between EAT volume and abnormal renal function remained significant (per SD cm^3^; OR, 1.78; 95% CI, 1.23–2.58; *P* = 0.002) in multivariate logistic regression analysis (adjusted for age, sex, BMI, body surface area, active smoking, hypertension, diabetes mellitus, dyslipidemia). Due to the population selection bias in this study, a specially designed study is needed to determine the relationship between EAT volume and renal dysfunction. Interestingly, A few studies indicated that EAT could be modified by intense lifestyle modification and lipid-lowering therapy (43, 44). Besides, two randomized controlled trials by Iacobellis et al. demonstrated that both liraglutide [an analog of glucagon-like peptide 1 (GLP-1)] and dapagliflozin [a selective sodium-glucose cotransporter 2 inhibitor (SGLT-2i)] significantly and rapidly reduced EAT thickness (45, 46). Cardiovascular protective effects of GLP-1 receptor agonists and SGLT-2i may be mediated by EAT. In this case, EAT may be a novel therapeutic target. Further research is warranted to assess its therapeutic potential.

Moreover, we demonstrated that EAT volume was significantly and positively correlated with the presence of hemodynamically significant CAD independently of conventional risks and CACs. Likewise, a retrospective study of 270 suspected CAD patients by Nappi et al. indicated that EAT volume was independently related to impaired myocardial perfusion according to myocardial perfusion reserve obtained by positron emission tomography/computed tomography (PET/CT) (47). Recently, Yu et al. showed a significant association between EAT volume and myocardial ischemia based on single-photon emission computed tomography myocardial perfusion imaging (SPECT-MPI) (48). Besides, Xie et al. reported the connection between EAT volume and lesion-specific ischemia derived from CT-FFR (49). In line with these prior studies, we demonstrated that EAT volume was related to myocardial ischemia. Unlike previous studies, we used QFR as the reference to identify hemodynamically significant coronary lesions. QFR was automatically calculated according to coronary angiographic images, which could provide a more accurate anatomical assessment of coronary arteries than computed tomography images by avoiding the artifacts caused by high heart rate, breath and beam hardening (50). Thus, QFR has been shown to be a highly accurate diagnostic tool compared to invasive FFR (14). Moreover, we performed the study on the individual level rather than on the vessels level and measured the QFR of major coronary arteries in all patients. QFR_min_ ≤0.8 was used to determine the presence of hemodynamically significant CAD, while the number of vessels with QFR ≤0.8 and QFR_min_ were used to quantify the severity of hemodynamically significant CAD. Our results indicated that the number of vessels with QFR ≤0.8 increased across tertiles of EAT volume and was directly correlated with EAT volume (*R*, 0.34; *P *< 0.001). EAT volume was negatively associated with QFR_min_ in a significant way regardless of traditional risks and CACs. It appears that hemodynamically significant CAD severity is independently and positively associated with EAT volume. Yet, a small cross-sectional study of 38 patients by Muthalaly et al. reported myocardial CT perfusion (CTP) imaging or invasive FFR analysis did not show a link between EAT volume and perfusion defects or hemodynamically significant coronary stenosis (51). Notably, Wen et al. found a strong correlation between hemodynamically significant coronary stenosis and pericoronary adipose tissue (PCAT) CT attenuation rather than volume (52). The controversial results among studies might be partly attributed to different sample sizes, ethnic populations and experimental protocols. Large prospective multicenter studies are needed to further determine the connection between EAT and coronary physiology.

Furthermore, we evaluated the ability of EAT volume to detect hemodynamically significant CAD. EAT volume was found to be a strong indicator of hemodynamically significant CAD, and its combination with CACs improved the prediction. Consistently, Xie et al. discovered that EAT volume coupled with clinical data improved the predictive accuracy of lesion-specific ischemia (49). Brandt et al. also found that the combined method of EAT volume with plaque quantification is a similar predictor of lesion-specific ischemia as CT-FFR (32). An earlier study demonstrated that obstructive CAD is highly correlated with myocardial ischemia when invasive FFR is used as a reference (36). Here, we found that the combination of EAT volume with obstructive CAD significantly improved diagnostic performance for hemodynamically significant CADs, which may help guide more effective revascularization strategy and improve clinical outcomes. Through noninvasive imaging methods (non-contrast CT or CCTA), EAT volume can be routinely acquired, which makes it competitive in the clinical setting. Generally, EAT volume may improve risk stratification for CAD patients and their clinical outcomes.

There are several limitations to our study. Firstly, this is a retrospective study that can only investigate association but not causation. Secondly, the sample size of this study is not large enough, which may lead to a selection bias. Thus, Large prospective multicenter studies will be necessary to validate our results. Thirdly, the determination of hemodynamically significant CAD was evaluated using QFR instead of invasive FFR. However, based on invasive FFR, QFR has excellent diagnostic accuracy. Fourthly, our study cannot confirm prognostic implications due to the absence of event follow-up. Fifthly, we only investigated the relationship between EAT volume and hemodynamically significant CAD. According to previous studies, location-specific EAT thickness may be a better biomarker of CAD than EAT volume (22, 23). Further research is needed to explore the effect of location-specific EAT thickness on myocardial ischemia. Therefore, these will be the direction of our future research.

In conclusion, this study demonstrated that EAT volume was significantly and positively correlated with the presence and severity of hemodynamically significant CAD in Chinese patients with known or suspected CAD, independent of conventional risks and CACs. In comparison with obstructive CAD alone, EAT volume assistance improved diagnostic performance for hemodynamically significant CAD. Therefore, EAT may be a credible noninvasive marker of hemodynamically significant CAD and may improve clinical outcomes and risk stratification.

## Data Availability

The raw data supporting the conclusions of this article will be made available by the authors, without undue reservation.
