# Supplementary material for: Impact of epicardial adipose tissue volume on hemodynamically significant coronary artery disease in Chinese patients with known or suspected coronary artery disease
Source: Front Cardiovasc Med. 2023 Mar 21;10:1088961. doi: 10.3389/fcvm.2023.1088961 (PMC10071511; doi:10.3389/fcvm.2023.1088961)
Supplement: Supplementary file 1 [file Table1.docx]

Supplementary Material

# Supplementary Figures


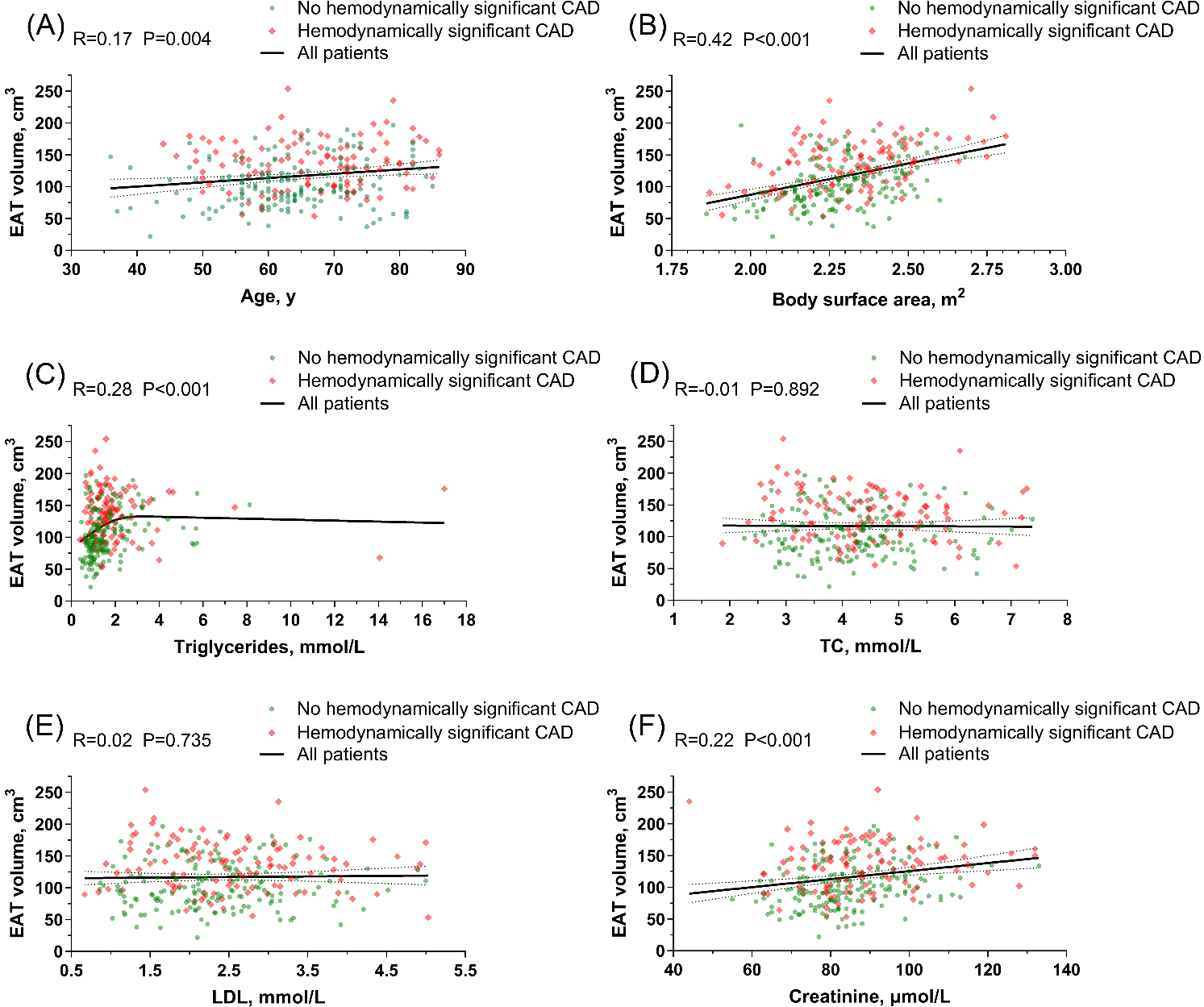


**Supplementary Figure 1. Association of EAT volume with age, body surface area, triglycerides, TC, LDL and creatinine. (A)** Correlation between EAT volume and age. **(B)** Correlation between EAT volume and body surface area. **(C)** Correlation between EAT volume and triglycerides. **(D)** Correlation between EAT volume and TC. **(E)** Correlation between EAT volume and LDL. **(F)** Correlation between EAT volume and creatinine.

CAD, coronary artery disease; EAT, epicardial adipose tissue; LDL, low-density lipoprotein; TC, total cholesterol.


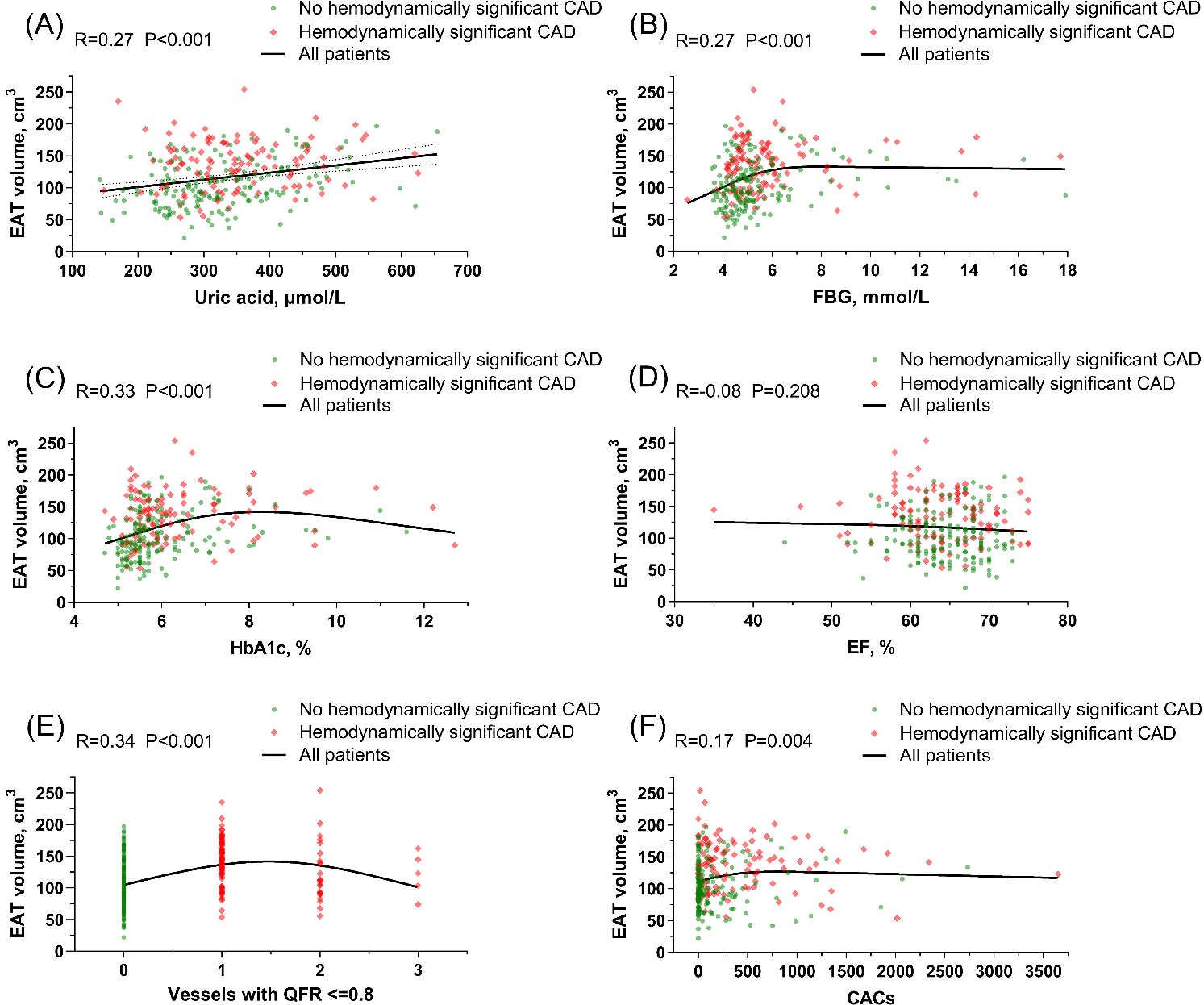


**Supplementary Figure 2. Association of EAT volume with uric acid, FBG, HbA1c, EF, CACs and the number of vessels with QFR ≤0.8. (A)** Correlation between EAT volume and uric acid. **(B)** Correlation between EAT volume and FBG. **(C)** Correlation between EAT volume and HbA1c. **(D)** Correlation between EAT volume and EF. **(E)** Correlation between EAT volume and the number of vessels with QFR ≤0.8. **(F)** Correlation between EAT volume and CACs.

CACs, coronary artery calcium score; CAD, coronary artery disease; EAT, epicardial adipose tissue; EF, ejection fraction; FBG, fasting blood glucose; HbA1c, glycated hemoglobin; QFR, quantitative flow ratio.
